# Changes of total protein and protein fractions in broiler chickens during the fattening period

**DOI:** 10.14202/vetworld.2019.598-604

**Published:** 2019-04-23

**Authors:** Csilla Tóthová, Edina Sesztáková, Bohumil Bielik, Oskar Nagy

**Affiliations:** 1 Clinic of Ruminants, University of Veterinary Medicine and Pharmacy in Košice, Košice, Slovak Republic; 2 Clinic of Birds, Exotic and Free Living Animals, University of Veterinary Medicine and Pharmacy in Košice, Košice, Slovak Republic

**Keywords:** broilers, electrophoresis, fattening, protein fractions, serum

## Abstract

**Background and Aim:**

Blood proteins in birds serve as an important indicator in the evaluation of health status and represent a basis in general biochemistry allowing the identification of metabolic alterations. The objective of this study was to evaluate the protein profile in broiler chickens extended by the concentrations of serum protein fractions at different periods of fattening.

**Materials and Methods:**

Into the evaluation, we included 24 clinically healthy Ross 308 line meat-type chickens at the age of 2 days. Blood samples were taken on day 4, 18, 32, and 46 of fattening always from six randomly selected chickens. Chickens were fed with a commercial starter, grower, and finisher feeds. The concentrations of total serum protein and protein fractions were evaluated.

**Results:**

Various significant changes in the proportion of the individual protein fractions were found during the observed period except for the beta-globulins in all protein fractions and the albumin/globulin (A/G) ratio. At the beginning of the fattening period, the relative concentrations of albumin, α_1_-globulins, and A/G ratio were significantly lower and the values of α_2_- and γ-globulins significantly higher (p<0.05). The values of pre-albumin fraction were found as a small band preceding the albumin fraction differed significantly between the different age groups of chickens (p<0.05). The total serum protein concentrations showed higher values in older broilers; the significantly highest mean value was recorded on day 32 of fattening.

**Conclusion:**

The results suggest that fattening and age of broilers influences not only the production patterns, metabolic processes, and lipid and mineral profile but also the parameters of protein profile. However, seeing that some contradictory data exist regarding the number and size of globulin fractions in chickens, further analyses are needed.

## Introduction

Blood proteins in birds serve as an important indicator in the evaluation of health status, but also of production features, and their evaluation represents a basis in general biochemistry allowing the identification of metabolic alterations [1,2]. Seeing that the blood proteins have numerous physiological roles in the body and the maintenance of homeostasis, the determination of their concentrations in avian patients is of exceptional significance for the evaluation of health state, as well as body condition [3]. Taking into consideration the basic anatomical and functional differences between birds and mammals, the values of blood proteins may be markedly different in the species. The total protein (TP) concentrations in birds are about the half of values (approximately 40 g/l) measured in mammals, caused by the presence of extremely high blood concentrations of osmotically active glucose, which may reduce the protein concentration to maintain the colloid osmotic pressure [4]. Electrophoresis is one of the most important methods to evaluate any changes in serum protein concentrations, especially in cases of hyperproteinemia describing the distribution of protein fractions and distinguishing polyclonal (inflammatory) increase of gamma-globulins from monoclonal caused principally by the overproduction of a single immunoglobulin [5,6].

Among various pathological conditions causing changes in the serum biochemistry values, there are several physiological (breeding, molting, and nest building), as well as exogenous factors (husbandry conditions), that may potentially affect the concentrations of serum proteins also in birds [7,8]. The process of growth and development in chickens, especially in broilers, is a very intensive period accompanied by great metabolic changes, increase of body mass, and accumulation of enormous amount of muscle in a very short time [9]. Thus, age and the associated production processes in growing poultry or meat-type domestic birds are other important factors that may influence the intensity of metabolism and induce changes in the pattern of serum proteins. Similarly, to some mammalian species, avian neonates and juveniles may show age-related differences in serum proteins, being lower in young animals compared with adult ones. Some studies were carried out to determine the serum proteins and their changes in broilers at different ages [2,10]. However, these studies were focused on the examining of differences in serum protein concentrations, protein fractions, or several serum proteins in the later period of growth, especially in chickens from the 14^th^ to 42^nd^ days of age. The possible changes in the concentrations of serum proteins in the earlier stages of development are to a lesser extent documented.

Since there are little data on serum protein fractions in broiler chickens, in general, as well as in relation to younger ones, the study was aimed besides the evaluation of changes in TP also at the quantification of changes in serum protein fractions using agarose gel electrophoresis at different periods of fattening (from 4 to 46 days of age).

## Materials and Methods

### Ethical approval

All procedures with animals in the study were conducted in accordance with national or institutional guidelines for the care and use of animals and the ethical standards of the institution or practice. Blood samples were collected as per standard sampling procedure without any harm to the animals. The approval from the Institutional Animal Ethics Committee was not required; the study did not affect the normal animal physiology.

### Animals and blood sample collection

The evaluation was performed on 24 clinically healthy Ross 308 line fattening chickens from a large-scale chicken farm. They were included in the study at the age of 2 days. After delivery the chickens to the Clinic of Birds, Exotic and Free Living Animals of the University of Veterinary Medicine and Pharmacy in Košice (Slovak Republic), they were placed into a litter-floored wire cage at the temperature of 32°C. This was gradually reduced with the age of chickens to 24°C over the evaluated 6 weeks, with continuous lighting 24 h a day during the 1^st^ week of life and then 16 h a day until the end of evaluation. Chickens were fed up to the day 14 of age with a commercial starter feed Broiler Mini Forte, from the day 15 to the day 35 of age with the grower feed Broiler Midi Forte, and from day 36 to the end of fattening period with a commercial finisher feed Broiler maxi (Energys Hobby, De Heus, Czech Republic). The main chemical composition of the diets and additives in the diet is presented in Table-1. The chickens were fed *ad libitum* with free access to water. The health status of the animals was evaluated daily until the end of the study using routine diagnostic procedures and was oriented to the observation of general health state, feed intake, weight gain, and behavior. The evaluated chickens were in good general health without any obvious clinical signs of diseases. The body weights of the chickens were measured before any sample collection. The average body weight on the days of blood collection was 67.3±9.7 g, 441.3±97.1 g, 1514.0±102.6 g, and 2956.0±271.7 g.

**Table-1:** Chemical composition of the used broiler diets and additives in the diets.

Composition of feed in 1 kg of dry matter	Units	Type of broiler feed		

		Starter	Grower	Finisher
Crude protein	%	19.9	18.9	17.7
Crude fiber	%	3.0	3.5	3.8
Oil and fat	%	3.9	4.1	4.7
ME	MJ	11.5	11.7	12.0
Ash	%	6.3	4.3	3.7
Lysine	%	1.11	1.09	1.01
Methionine	%	0.49	0.47	0.43
Ca	%	0.9	0.6	0.40
P	%	0.72	0.44	0.43
Na	%	0.14	0.13	0.12
Vitamin A	IU/kg	10,500	8000	6500
Vitamin E	mg/kg	70	20	16
Salinomycin	mg/kg	70	70	0

ME= Metabolizable energy

Blood samples were obtained in various stages of fattening period. The first blood sample was taken from six randomly selected chickens on the day 4 of fattening and then on days 18, 32, and 46 of fattening always from another randomly selected six chickens in each group. Chickens at the age of 4 and 18 days were blood from the jugular vein, while blood samples from chickens at the age of 32 and 46 days were collected from the wing vein into serum gel separator tubes without additives and anticoagulants (Sarstedt, Nümbrecht, Germany). Serum was separated after letting blood samples to coagulate at room temperature and centrifugation at 4000 g for 15 min. The serum was separated from the clot and dispensed into plastic tubes, stored was in a freezer at −20°C until the analysis.

### Laboratory analysis

To evaluate the changes in the protein profile during the fattening period in chickens, serum samples were analyzed for the concentrations of TP and main protein fractions. The TP (g/l) were determined using an automated biochemical analyzer Alizé (Lisabio, France) according to the biuret method with commercially available diagnostic kits (Randox, Crumlin, United Kingdom). The serum protein fractions were separated by zone electrophoresis on an agarose gel using an automated electrophoresis system Hydrasys (Sebia Corporate, France) and commercial diagnostic kits Hydragel 7 Proteine (Sebia Corporate, France) according to the procedure described by the manufacturer. The densitometry scanning system Epson Perfection V700 (Epson America Inc., USA) was used to scan the electrophoretic gels based on the method of light transmission and conversion into an optical density curve. The gel images were visualized using the computer software Phoresis version 5.50 (Sebia Corporate, France). The following protein fractions were identified: Pre-albumin, albumin, alfa_1_- (α_1_-), alfa_2_- (α_2_-)_,_ beta- (β-), and gamma (γ-)-globulins. Each protein fraction was expressed as relative concentrations (%) according to the obtained optical density. The absolute concentrations (g/l) of the fractions were quantified consequently from the total serum protein concentrations. The ratio of albumin to globulins (A/G) was calculated by dividing the sum of pre-albumin and albumin by the sum of globulin fractions.

### Statistical analysis

The statistical analyses of the data were processed using the GraphPad Prism V5.02 programme (GraphPad Software Inc., California, USA). Descriptive statistical procedures were used to calculate arithmetic means (x) and standard errors of means for each variable and sample collection time. Kolmogorov–Smirnov test for normality was used for analysis of the distribution of data. Analysis of variance test was applied to examine the changes in the concentrations of evaluated parameters during the fattening period. The significance of differences in values between the sample collections was evaluated by Tukey’s multiple comparisons test. Significance was considered at 5% probability level.

## Results

**Figure-1:**
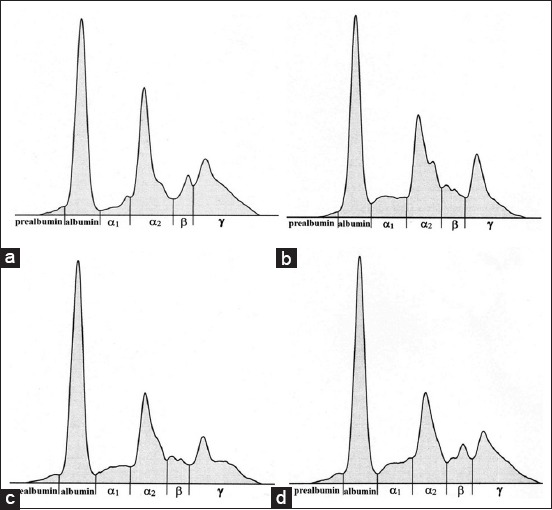
(a-d) Representative electrophoretograms from broilers showing the protein fractionation into six fractions: Pre-albumin, albumin, α_1_-, α_2_-, β-, and γ-globulins at different age groups: (a) day 4; (b) day 18; (c) day 32, and (d) day 46.

The data obtained in different age groups of chickens evaluated during the fattening period are presented in Tables-2 and 3. Representative examples of electrophoretograms in different age groups of broilers are presented in Figure-1.

**Table-2:** Changes in the relative concentrations of serum protein fractions (%) and albumin/globulin ratio (A/G) in broiler chickens during the fattening period (mean±SE).

Variables	Age of chickens				p-value

	Day 4	Day 18	Day 32	Day 46	
Pre-albumin	1.67±0.18^a,b^	1.17±0.17^a^	1.45±0.10^a,b^	2.03±0.22^b^	<0.05
Albumin	33.6±0.74^a^	36.7±1.31^a,b^	39.6±1.82^b^	39.8±1.64^b^	<0.05
a_1_-globulins	5.2±0.46^a^	8.1±0.71^b^	7.1±0.22 ^a,b^	7.6±0.62^b^	<0.01
a_2_-globulins	30.3±0.72^a^	28.4±0.88^a^	22.9±0.78^b^	24.2±0.60^b^	<0.001
b-globulins	7.3±0.39	8.0±0.48	12.1±2.57	7.6±0.70	n.s
g-globulins	22.0±1.26^a^	17.7±0.89^b^	16.8±0.75^b^	18.9±1.04^a,b^	<0.01
A/G	0.59±0.03^a^	0.68±0.05^a,b^	0.81±0.08^a,b^	0.85±0.08^b^	<0.05

p-value–significance of the ANOVA test, n.s=Not significant, SE=Standard error of mean,

a,b =Different superscripts in rows mean statistically significant difference between the sample collections (Tukey’s test, level of significance–p<0.05)

**Table-3:** Changes in the absolute concentrations of total serum protein and protein fractions (g/l) in broiler chickens during the fattening period (mean±SE).

Variables	Age of chickens				p-value

	Day 4	Day 18	Day 32	Day 46	
TP	28.0±1.21^a^	31.0±1.07^a,b^	33.2±1.89^b^	29.2±0.68^a,b^	<0.05
Pre-albumin	0.45±0.03^a,b^	0.37±0.04^a^	0.48±0.03^a,b^	0.58±0.07^b^	<0.05
Albumin	9.4±0.45^a^	11.3±0.39^b^	13.0±0.39^b^	11.6±0.61^b^	<0.001
a_1_-globulins	1.5±0.12^a^	2.5±0.28^b^	2.4±0.19^b^	2.2±0.21^a,b^	<0.01
a_2_-globulins	8.5±0.48^a^	8.8±0.32^a^	7.6±0.29^a,b^	7.0±0.16^b^	<0.01
b- globulins	2.1±0.15	2.5±0.20	4.3±1.15	2.2±0.21	n.s
g- globulins	6.2±0.43	5.5±0.42	5.5±0.21	5.5±0.29	n.s

p-value–significance of the ANOVA test, n.s=Not significant, SE=Standard error of mean,

a,b =Different superscripts in rows mean statistically significant difference between the sample collections (Tukey’s test, level of significance–p<0.05), TP=Total protein

The pre-albumin fraction was visible as a small band preceding the main albumin fraction. Its relative concentrations differed significantly between the different age groups of chickens (p<0.05), with the lowest mean value in broilers at the age of 18 days and the highest on day 46 of fattening (Table-2). The relative concentrations of albumin showed a gradual and significant increase in values with age (p<0.05). A significantly higher proportion of albumin fraction was recorded at the age of 32 and 46 days (p<0.05). Significant differences between the evaluated age groups of chickens were also observed in the α_1_-globulin fraction (p<0.01). The lowest mean value was found in chickens at the age of 4 days; the values obtained in older chickens were significantly higher (p<0.05). Opposite changes and significantly lower values during the fattening period were observed in the relative concentrations of α_2_- and γ-globulins (p<0.001 and p<0.01, respectively).

On the other hand, the relative values of β-globulin fractions and A/G ratios showed a tendency of higher values in older chickens. The non-significantly highest values of β-globulins were detected on the day 32 significantly highest A/G ratios on day 46 of fattening (p<0.05). In chickens from the day 18 of fattening, the β-globulin fraction tended to divide into two subfractions (Figure-1).

The concentrations of TP in chickens revealed a tendency of the significant gradual increase until the day 32 of fattening (p<0.05), the values in samples on day 46 of fattening were slightly lower (Table-3). Significant differences between the age groups of chickens were observed in absolute concentrations of pre-albumin (p<0.05), with lower values in younger broilers compared with older ones. The absolute concentrations of albumin during the fattening period showed significant changes (p<0.001). The mean concentration of albumin on day 4 was significantly lower than the means found in older chickens (p<0.05). Significant changes were also found in the concentrations of α_1_- and α_2_-globulin fractions (p<0.01). The values of α_1_-globulins recorded on days 18 and 32 of fattening were significantly higher (p<0.05) compared with those sampled on day 4 of age. The concentrations of α_2_-globulin fraction on days 4 and 18 of fattening were approximately uniform, but the values obtained on day 46 were significantly lower (p<0.05) than in younger broilers. In the absolute concentrations of β-globulins, a tendency of no significant gradual increase of values until day 32 of fattening was observed. At the end of the fattening, the mean concentration of β-globulins was lower and similar to 4-day-old chickens. The highest mean value of γ-globulin concentrations was found in chickens at the beginning of fattening on day 4. The mean values recorded in older chickens were lower.

## Discussion

Several physiological and pathological factors have been investigated to describe possible qualitative and quantitative alterations in the concentrations of blood proteins, reflecting the actual general health state and condition of the evaluated animals, including bird species [8]. These variations are more dynamic in young animals and usually are related to intensive metabolic processes and changes in nutrition. The results presented in our study showed in broiler chickens a tendency of gradual increase of TP concentrations, being the highest in chickens on day 32 of fattening. The results of Filipović *et al*. [2] and Piotrowska *et al*. [3] showed in chickens, a significant increase of serum protein concentrations from the 14^th^ to the 42^nd^ days of age, which was related to the growth processes and feeding with protein-rich diets during the fattening period. This period is characterized by extensive supply of amino acids for very intensive somatic growth, and thus, the liver increases the synthesis of serum proteins resulting in the increase of the concentrations of TP in the blood [10]. In our study, the tendency of a gradual increase of TP concentrations until day 32 of fattening period was followed by a no significant decrease in values in chickens at the age of 46 days. This may be associated with changes in nutrition (transition from grower to finisher diet at the age of 36 days), different diet composition (lower content of crude protein in the finisher diet in comparison with the protein-rich starter and grower diets), and food consumption during growth, as well as the metabolic rate and physical condition of the growing chickens. Furthermore, the intensity of the building of proteins into the tissues may significantly influence the concentrations of proteins in the blood, as well as their composition [4]. This factor is very important in chickens due to the extremely rapid accumulation of body mass during the relatively short time of fattening.

In some avian species, a pre-albumin fraction may be present as a distinct band preceding the main albumin fraction on the electrophoretogram, but its type and size vary between different bird species and according to the used electrophoretic medium [8]. Filipović *et al*. [2] did not observe the presence of pre-albumin fraction in broiler chickens. In our study, pre-albumin was in chickens visualized as a band anodic to the albumin fraction using agarose gel electrophoresis and its concentrations varied significantly among the different age groups of chickens with the highest mean relative value in those at the end of fattening (day 46). In chickens, the influence of age on the concentrations of pre-albumin during the growth is not described, but it seems that, similarly to mammals, its serum concentrations are associated with the recent nutritional status and reflect the balance between protein synthesis and degradation [8,11]. Regarding the concentrations of albumin, our results showed a trend of gradual increase with age being the highest on days 32 and 46 of fattening, thus helping to maintain the metabolic balance during the period of rapid growth. Piotrowska *et al*. [3] also observed an increasing tendency of serum albumin in broiler chickens with the highest value (17.1 g/l) on the day 42 of fattening. Similar increase of albumin concentrations was found by Szabó *et al*. [10] in growing turkeys between 3 days and 8 weeks of age as a consequence of very quick and intensive somatic growth. Unlike these results, Filipovič *et al*. [2] recorded in chickens relatively stable values of albumin during the period from the 2^nd^ to 6^th^ weeks of age, probably caused by its rapid utilization by the synthesis of tissue proteins.

In the present study, marked variations between different age groups of chickens were also found in the concentrations of both α_1_- and α_2_-globulins. The concentrations of α_1_-globulins on days 18 and 32 of fattening were about 1.5-fold higher compared with those sampled on day 4 of fattening, while the values of α_2_-globulins were relatively constant at the age of 4 and 18 days with a tendency of more marked decrease at the end of fattening. Only Filipović *et al*. [2] presented a study dealing with the evaluation of several globulin fractions in chickens during fattening, and they recorded approximately a 3-fold increase of α_1_-globulins on the 28^th^ day of fattening with a significant decrease of values on day 42 of fattening. Furthermore, they observed gradually increasing concentrations of α_2_-globulins during the fattening period. However, the α_2_-globulin values measured by Filipović *et al*. [2] were markedly lower (2.91-3.99 g/l) compared with those presented in our study (7.0-8.5 g/l). The α-globulin fractions are composed (among others) of various lipoproteins including α_1_-, α_2_-lipoprotein, as well as pre-β-lipoprotein, which are primarily designated to the transport of lipid molecules in biological fluids [5,12]. Thus, higher concentrations of α-globulins in chickens on day 18 of fattening might be caused by the increased transport of fatty molecules in the body to supply great energy requirements for intensive metabolic reactions in this period and for their building into tissue structures [2].

Furthermore, many other diagnostically important proteins including acute phase proteins (α_1_-acid glycoprotein, ceruloplasmin, and serum amyloid A) migrate into the α-fractions [6]. However, increased concentrations of acute phase proteins are not always the consequence of the activated acute phase response since their values may also be elevated during the exposure of the organism to other stressors and changed environmental conditions [13]. Therefore, higher concentrations of α-globulins observed in chickens during some stages of fattening may be associated with quick growth, intensive metabolic processes, changes in nutrition, as well as various rearing factors. Takahashi *et al*. [14] also reported that the concentrations of α_1_-acid glycoprotein in broilers might be influenced by the age and nutritional changes. Similarly, O’Reilly *et al*. [15] observed in broiler breeding lines significant age-related changes in the concentrations of serum amyloid A and ceruloplasmin. Furthermore, serum amyloid A is involved in the modulation of lipoprotein transport and metabolism, and after secretion into the bloodstream, it associates with high-density lipoprotein molecules [16], which may also alter the concentrations of α-globulins in chickens during fattening.

In the concentrations of β-globulins, tendency of a gradual increase was observed with the highest mean values in chickens on day 32 of fattening. Similarly, Filipović *et al*. [2] reported the increase of β-globulins in broilers during fattening. However, the results of their study showed the separation of β-globulins into β_1_- and β_2_-globulin fractions and both of them increased in the second and third period of fattening. Transferrin, ovotransferrin, fibronectin, and hemopexin were identified in the β-globulin fraction [17]. Transferrins belong to the group of iron-binding glycoproteins, which are in chickens produced by the liver as serotransferrin and by the oviduct as ovotransferrin [18]. Both are involved in iron metabolism, transport, and delivery [19]. Very similar functions were ascribed to ferritin, which is a transport protein that binds to iron, stores it in a bioavailable and non-toxic form limiting, thus the supply of free iron [20]. Ferritin plays a crucial role also in the detoxification of harmful excess of toxic low-molecular-weight cellular iron compounds [21]. The extremely rapid growth, intensive erythropoiesis, and hemoglobin synthesis in chickens are associated with excessive need for iron, which may be particularly responsible for increased production of β-globulins during this period [2]. Furthermore, the β-fraction contains also components C3 and C4 of complement, which may be involved in the environmental stress response [7,22]. Seeing that chickens are exposed to many environmental stimuli and stressful conditions during fattening, higher concentrations of β-globulins also observed in our study may be explained by these influences.

The γ-zone contains predominantly immunoglobulins. Some immunoglobulin classes (IgM and IgG) may migrate in the β-globulin fraction, but the majority of immunoglobulins migrate in the γ-zone [23]. In chickens, three classes of immunoglobulins were isolated: IgY (the avian equivalent to mammalian IgG), IgA, and IgM [24]. The IgY antibody isotypes are transferred from the dams to their offsprings, which have to protect the newly hatched chicks from the pathogens [25]. In the study presented by Filipović *et al*. [2], a significant increase of γ-globulin concentrations was observed with aging, which was probably related to the maturation of immune system. In contrast to these results, the highest mean γ-globulin concentrations in our study were found in chickens at the age of 4 days. The relative values measured in chickens on days 18 and 32 of fattening showed a trend of significant decrease that could be explained by the catabolism of maternal IgY in chicks [26]. Approximately by the day 21, the chicks start to synthesize own IgY [24], which may be observable also in our study in chickens sampled on day 46 of fattening through higher relative concentrations of γ-globulins compared with those obtained on days 18 and 32 of age. The changed proportion of albumin and globulins in the evaluated age groups of chickens was reflected in the changed A/G ratio with the lowest value in broilers sampled on day 4 of age and the highest in those sampled on day 46 of fattening. This reflects the higher concentrations of albumin to maintain the metabolic balance during this period of production.

## Conclusion

The evaluation of the TP and electrophoretic protein fractions in rapidly growing broiler chickens revealed several significant changes in their concentrations during the period of fattening. While the albumin, α_1_- and β-globulin fractions, and A/G ratios were the lowest in 4-day-old chickens and the values increased with age, the opposite dynamics were observed for α_2_- and γ-globulins. Pre-albumin concentrations were the lowest on day 18, and then the values increased and were significantly the highest at the end of the fattening. Changes in the concentrations of the analyzed individual protein fractions were reflected in the observed changes of TP concentrations with an increase of the values in older broilers. These results suggest that fattening and age of broilers influence not only the production patterns, metabolic processes, and lipid and mineral profile but also the parameters of protein profile. The evaluation of the serum protein electrophoretic pattern in birds is a difficult and complex issue, and the interpretation of the protein electrophoretograms requires some experiences in this field. It is important to take into account not only the evaluated avian species but also the age, physiological state, as well as production parameters of the investigated animal. Moreover, the correct interpretation of the results of protein electrophoresis is possible only in the context of the clinical picture and actual health status of the evaluated bird. However, seeing that some contradictory data exist regarding the number and size of globulin fractions in chickens, further analyses are needed.

## Authors’ Contributions

ON: Conceived and designed the study. ES and BB: Collected samples and performed the experiments. CT: Performed the laboratory analyses. CT and ON: Performed the statistical analyses. CT: Drafted the manuscript. ON: Revised the manuscript critically. All authors read and approved the final manuscript.
